# Supplementary material for: A tetravalent live attenuated dengue virus vaccine stimulates balanced immunity to multiple serotypes in humans
Source: Nat Commun. 2021 Feb 17;12:1102. doi: 10.1038/s41467-021-21384-0 (PMC7889627; doi:10.1038/s41467-021-21384-0)
Supplement: Supplementary file 1 — Supplementary Information [file 41467_2021_21384_MOESM1_ESM.pdf]

Supplementary Figure 1

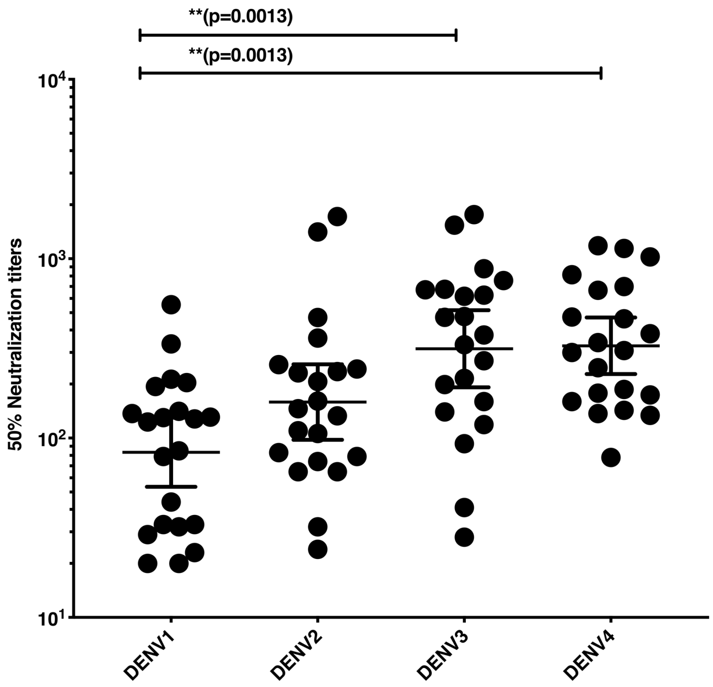

Supplementary Figure 1. Neutralizing antibody levels in TV003 recipients who were DENV naïve before vaccination. Neutralizing antibodies were measured in samples collected from 21(n=21) subjects 6 months after single dose of vaccination with TV003. Each data point represents the mean $Neut_{50}$ titer per individual for each serotype. Data were analyzed from a total number of 21 subjects indicated as dots from a single independent experiment and expressed as geometric mean titers ± 95% confidence intervals of the grouped samples. Nonparametric One-way Anova with Friedman Dunn's multiple comparisons test was used. Each p value was adjusted to account for multiple comparisons. (*****p<0.0001*; ****p<0.001; **p<0.01; *p<0.05).* The serotypes with significantly high geometric mean titers compared to other serotypes is shown (DENV3: **p= 0.0013; CI:288.2-707; DENV4: **p=0.0013; CI: 281.4-598.1).

Supplementary Figure 2

a

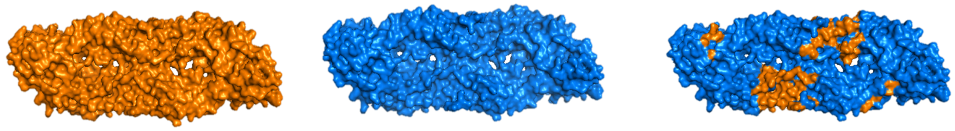

b

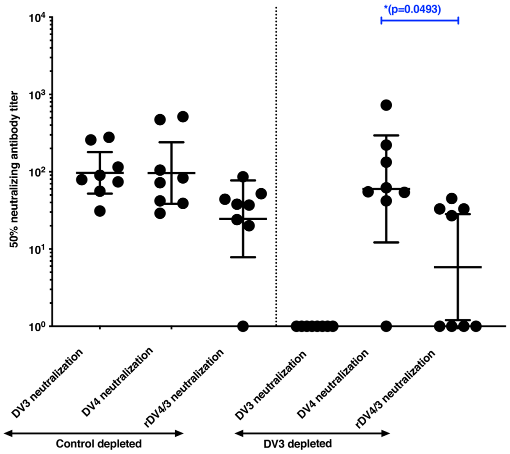

Supplementary Figure 2: TV003 induce varying levels of DENV4 serotype specific responses against known DENV4 neutralizing epitopes. a DENV3 wt (orange), DENV4 wt (blue) rDENV4/3 with DENV3 specific 5J7 mAb epitope transplanted into DENV4 (shown in orange) are depicted. It is the same region where the epitopes of DENV4-126, 131 nAb epitopes reside on DENV4. **b** Panel of 8 (n=8) TV003 sera with evidence of DENV4 TS responses were subjected to depletions against DENV3 to map the DENV4 TS nAb epitopes using the rDENV4/3 virus. This is a loss of function virus. Figure shows the $Neut_{50}$ titers for the BSA depleted and the DENV3 depleted serum samples against DENV3 wt, DENV4 wt and the rDENV4/3 viruses. Data were analyzed from a total number of 8 subjects indicated as dots from a single independent experiment and expressed as geometric mean titers ± 95% confidence intervals. Nonparametric One-way Anova with Friedman Dunn's multiple comparisons test **(b)** was used for the epitope mapping data to determine the loss or gain in neutralization to the recombinant chimeric viruses compared to the wild type parental backbone viruses. Each p value was adjusted to account for multiple comparisons. (*****p<0.0001*; ****p<0.001; **p<0.01; *p<0.05).* The significant loss in neutralization of DENV4 TS nAbs against rDENV4/3 is indicated (*p=0.0493; CI:2.2–33.2) **(b)**.

Supplementary Figure 3

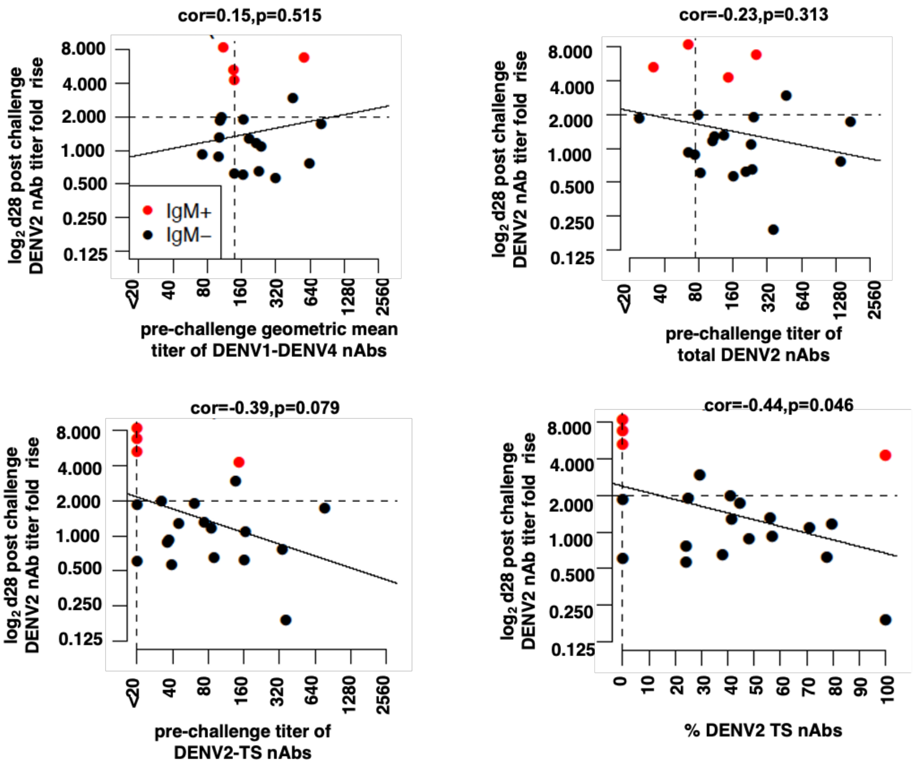

Supplementary Figure 3: Immune correlate analysis of DENV2 Ab level vs specificity. Figure shows the relationship of various predictors (total DENV2 nAb titer, absolute TS DENV2 nAb titer, DENV2 %TS responses, geometric mean nAb titers against DENV1-DENV4) in all the vaccine recipients (n=21) against DENV2 specific day 28 post challenge boost data. Points are colored by IgM positivity measured at day 28. Black solid line shows linear relationship between predictor and outcome variables. "Cor" indicates the Pearson's product-moment correlation and p-value. Odds ratio with 95% confidence intervals are shown for risk of ≥2-fold boost (equal to or above the horizontal dotted line) for those with predictor values greater than or equal to the titer threshold (indicated by vertical dotted line). Odds ratio confidence intervals that do not include 1 are significant. Data were analyzed by an exploratory statistical analysis to evaluate the relationship between pre-challenge nAb titer and boost in DENV2 nAb titer post-challenge. The correlation between predictor and outcome variables were evaluated by using Pearson's product-moment correlation. The odds ratio analysis of ≥2-fold boost in DENV2 nAb titer for those with predictor nAb levels above vs. at or below a specified threshold were estimated using the mosaic package in R.

Supplementary Table 1. DENV neutralizing antibody levels in subjects who received TV003 vaccine or placebo

	Subject	Reciprocal of $Neut_{50}$ titers against DENV1-4			
		DENV1	DENV2	DENV3	DENV4
TV003 vaccine	1	141	207	93	134
	2	123	235	270	341
	3	29	146	618	137
	4	23	65	332	300
	5	44	79	199	187
	6	213	106	672	143
	7	32	133	140	178
	8	85	74	215	78
	9	137	231	627	160
	10	33	24	376	382
	11	555	32	41	473
	12	<20	65	476	174
	13	79	243	160	247
	14	130	1411	756	1142
	15	33	83	880	308
	16	128	`470	675	1024
	17	204	161	471	667
	18	<20	361	28	814
	19	194	257	1761	1183
	20	335	1720	1541	463
	21	131	110	119	700
Placebo	22	<20	<20	<20	<20
	23	<20	<20	<20	<20
	24	<20	<20	<20	<20
	25	<20	<20	<20	<20

Supplementary table 2. Level of serotype specific neutralizing antibody responses in TV003 vaccine recipients

	Subject	Absolute serotype specific nAb titer >20 (% type-specific neutralizing nAbs against DENV1-4)			
		DENV1	DENV2	DENV3	DENV4
TV003 vaccine	1	141(100)	161(78)	93(100)	134(100)
	2	74(60)	89(38)	184(68)	65(19)
	3	0	146(100)	618(100)	103(75)
	4	0	0	139(42)	300(100)
	5	0	32(41)	165(83)	187(100)
	6	170(80)	85(80)	571(85)	89(62)
	7	0	74(56)	95(68)	142(80)
	8	57(70)	36(48)	142(66)	74(95)
	9	101(74)	164(71)	445(71)	146(91)
	10	0	0	323(86)	309(81)
	11	555(100)	0	0	407(86)
	12	0	37(57)	476(100)	129(74)
	13	44(56)	61(25)	83(52)	106(43)
	14	0	339(24)	76(10)	1085(95)
	15	0	0	0	308(100)
	16	22(17)	136(29)	675(100)	584(57)
	17	92(45)	39(24)	188(40)	213(32)
	18	0	361(100)	0	814(100)
	19	194(100)	0	722(41)	1183(100)
	20	235(70)	774(45)	1541(100)	139(30)
	21	88(67)	45(41)	75(63)	231(33)
Placebo	22	0	0	0	0
	23	0	0	0	0
	24	0	0	0	0
	25	0	0	0	0

Supplementary table 3. rDENVs used for epitope mapping studies

Epitope mapping	rDENV	Backbone serotype	Test serotype	Serotypes other than backbone and test serotypes
DENV1	rDENV2/1	DENV2	DENV1	DENV3,4
DENV2	rDENV4/2	DENV4	DENV2	DENV1,3
DENV3, DENV4	rDENV4/3	DENV4	DENV3	DENV1,2
DENV4	rDENV2/4	DENV2	DENV4	DENV1,3

Supplementary table 4. DENV2 neutralizing antibody boost and IgM data

Subject	6 months post vaccination DENV2 nAb titer	Day 28 DENV2 nAb titer post challenge	Fold rise in nAb against DENV2	DENV2 % TS	IgM increase post challenge (Y/N)
**1**	116	72	0.62	78	N
**2**	361	234	0.65	38	N
**3**	**263**	**1133**	**4.31**	**100**	**Y**
**4**	**303**	**2550**	**8.42**	**0**	**Y**
**5**	121	241	1.99	41	N
**6**	506	588	1.16	80	N
**7**	641	833	1.3	56	N
**8**	709	624	0.88	48	N
**9**	261	285	1.09	71	N
**10**	74	137	1.85	0	N
**11**	**83**	**444**	**5.35**	**0**	**Y**
**12**	60	56	0.93	57	N
**13**	299	566	1.89	25	N
**14**	1102	850	0.77	24	N
**15**	334	205	0.61	0	N
**16**	**315**	**931**	**2.96**	**29**	**N**
**17**	106	59	0.56	24	N
**18**	26	5	0.19	100	N
**19**	**430**	**2944**	**6.85**	**0**	**Y**
**20**	1413	2462	1.74	45	N
**21**	154	196	1.27	41	N

Bold indicates the individuals who showed a boost greater than 2-fold

Supplementary table 5: Comparison of the two depletion strategies (DENV1 and DENV234mix vs DENV2 and DENV134mix) in subjects in 6 and 19

Subject	Depletion Strategy	Absolute serotype specific nAb titer >20 (% type-specific neutralizing nAbs against DENV1-4)			
		DENV1	DENV2	DENV3	DENV4
6	DENV2 and DENV134 mix	170(80)	85(80)	571(85)	89(62)
	DENV1 and DENV234 mix	95(57)	157(69)	811(100)	168(61)
19	DENV2 and DENV134 mix	194(100)	0	722(41)	1183(100)
	DENV1 and DENV234 mix	167(88)	0	890(100)	777(88)
