# Supplementary material for: A tetravalent live attenuated dengue virus vaccine stimulates balanced immunity to multiple serotypes in humans
Source: Nat Commun. 2021 Feb 17;12:1102. doi: 10.1038/s41467-021-21384-0 (PMC7889627; doi:10.1038/s41467-021-21384-0)
Supplement: Supplementary file 3 — Reporting Summary [file 41467_2021_21384_MOESM3_ESM.pdf]

natureresearch

Corresponding author(s): Aravinda M.de Silva

Last updated by author(s): 7/16/2020

Reporting Summary

Nature Research wishes to improve the reproducibility of the work that we publish. This form provides structure for consistency and transparency in reporting. For further information on Nature Research policies, see Authors & Referees and the Editorial Policy Checklist.

Statistics

For all statistical analyses, confirm that the following items are present in the figure legend, table legend, main text, or Methods section.

n/a	Confirmed	
☐	☒	The exact sample size (n) for each experimental group/condition, given as a discrete number and unit of measurement
☐	☒	A statement on whether measurements were taken from distinct samples or whether the same sample was measured repeatedly
☐	☒	The statistical test(s) used AND whether they are one- or two-sided<br>*Only common tests should be described solely by name; describe more complex techniques in the Methods section.*
☒	☐	A description of all covariates tested
☒	☐	A description of any assumptions or corrections, such as tests of normality and adjustment for multiple comparisons
☐	☒	A full description of the statistical parameters including central tendency (e.g. means) or other basic estimates (e.g. regression coefficient) AND variation (e.g. standard deviation) or associated estimates of uncertainty (e.g. confidence intervals)
☒	☐	For null hypothesis testing, the test statistic (e.g. F, t, r) with confidence intervals, effect sizes, degrees of freedom and P value noted<br>*Give P values as exact values whenever suitable.*
☒	☐	For Bayesian analysis, information on the choice of priors and Markov chain Monte Carlo settings
☐	☐	For hierarchical and complex designs, identification of the appropriate level for tests and full reporting of outcomes
☐	☒	Estimates of effect sizes (e.g. Cohen's d, Pearson's r), indicating how they were calculated

Our web collection on statistics for biologists contains articles on many of the points above.

Software and code

Policy information about availability of computer code

Data collection	No computer code used
Data analysis	Data was graphed and analyzed using the GraphPad Prism software. We used the mosaic package in R to perform the odds ratio analysis.

For manuscripts utilizing custom algorithms or software that are central to the research but not yet described in published literature, software must be made available to editors/reviewers. We strongly encourage code deposition in a community repository (e.g. GitHub). See the Nature Research guidelines for submitting code & software for further information.

Data

Policy information about availability of data

All manuscripts must include a data availability statement. This statement should provide the following information, where applicable:

- Accession codes, unique identifiers, or web links for publicly available datasets
- A list of figures that have associated raw data
- A description of any restrictions on data availability

All data and analysis supporting the findings of this study are available within the paper.

Field-specific reporting

Please select the one below that is the best fit for your research. If you are not sure, read the appropriate sections before making your selection.

☒ Life sciences ☐ Behavioural & social sciences ☐ Ecological, evolutionary & environmental sciences

For a reference copy of the document with all sections, see nature.com/documents/nr-reporting-summary-flat.pdf

Life sciences study design

All studies must disclose on these points even when the disclosure is negative.

Sample size	We chose a sample size of (n=21) as the study reported here was mainly conducted to understand the plausible antibody correlates of protection in a phase 1human clinical trial conducted by NIH using the TV003 tetravalent live attenuated vaccine, which was a small efficacy study to understand protection of the vaccine against DENV2 challenge. In that study 21 subjects who were vaccinated were challenged with DENV2. Serum from the same 21 subjects was used in this study for our immune correlate analysis.
Data exclusions	No data were excluded
Replication	All samples were analyzed in duplicate within an experiment.
Randomization	All sera tested in these experiments came from healthy volunteers enrolled in a trial designed by the NIH to assess the protective efficacy of TV003 against DENV2.This was arandomized, double-blind, placebo-controlled trial.
Blinding	Authors were not blinded to group allocation in this study

Reporting for specific materials, systems and methods

We require information from authors about some types of materials, experimental systems and methods used in many studies. Here, indicate whether each material, system or method listed is relevant to your study. If you are not sure if a list item applies to your research, read the appropriate section before selecting a response.

Materials & experimental systems

n/a	Involved in the study	
☐	☒	Antibodies
☐	☒	Eukaryotic cell lines
☒	☐	Palaeontology
☒	☐	Animals and other organisms
☐	☒	Human research participants
☐	☒	Clinical data

Methods

n/a	Involved in the study	
☒	☐	ChIP-seq
☒	☐	Flow cytometry
☒	☐	MRI-based neuroimaging

Antibodies

Antibodies used	4G2, 2H2, 1M7, 2D22,3F9,4J23,5H2,126, 131,EDE1 C8, EDE2 B7
Validation	4G2 --> Henchal et al., 1985; Am J Trop Med Hyg<br>2H2--> Scott A. Smith et al ., 2016; Journal of virology<br>1M7 -->Scott A. Smith et al., 2014; Journal of Virology<br>2D22 --> Firbriansah et al., 2015; Science<br>3F9 --> Emily N. Gallichotte et al., 2018; Plos pathogens<br>4J23--> Scott A. Smith et al ., 2014; Journal of Virology<br>5H2--> Ching-Juh Lai et al., 2007; Journal of virology<br>126, 131--> Nivarthi et al., 2017, Journal of Virology<br>EDE1 C8, EDE2 B7 --> Rouvinsky et al., 2015; Nature<br>anti-human IgG-AP: Sigma (A9544)<br>anti-mouse IgG-HRP: KPL 074-1806

Eukaryotic cell lines

Policy information about cell lines

Cell line source(s)	Vero-81 cell lines. Purchased from ATCC (CCL-81), C636 ATCC (CRL-1660)
Authentication	Cell lines used were not authenticated after purchase
Mycoplasma contamination	Cell lines used are mycoplasma free
Commonly misidentified lines (See ICLAC register)	n/a

Human research participants

Policy information about studies involving human research participants

Population characteristics	A total of 48 flavivirus-naive subjects were enrolled 24 at Baltimore,MD and Burlington, VT. There were no statistically significant differences in the mean age (29.4 years versus 30.8 ± 1.7 years), gender (54.2% male versus 66.7% male), or race (58.3% White versus 62.5% White) in TV003 recipients compared to control recipients, respectively. Forty-one subjects returned for challenge with rDENV2delta30(21 TV003 recipients and 20 placebo recipients). There were no significant differences in the age, gender,or race between the two groups at challenge. A detailed description of the population characterstics is in the original clinical protocol
Recruitment	Subjects were recruited from a variety of sources including, but not limited to subjects previously enrolled in vaccine trials at the clinical sites, by the use of center wide IRB approved screening protocol. After an initial phone screen (Using IRB approved Phone screen/initial contact form) by clinic staff focused on providing background information of the trial and a review of basic inclusion and exclusion criteria a screening visit was scheduled. Each subject read the consent form, was encouraged to ask questions, and then completed a comprehension assessment. Informed consent was obtained in accordance with federal and international regulations (21CFR50 andICHE6). External independent monitoring was performed, and the National Institute of Allergy and Infectious Diseases Data Safety Monitoring Board reviewed all safety data every 6 months.<br><br>Detailed recruitment details can be found on page 46 in the original clinical protocol.
Ethics oversight	The studies were performed under an investigational new drug application reviewed by the U.S. Food and Drug Administration and approved by the Institutional review boards at the University of Vermont and Johns Hopkins University.

Note that full information on the approval of the study protocol must also be provided in the manuscript.

Clinical data

Policy information about clinical studies

All manuscripts should comply with the ICMJE guidelines for publication of clinical research and a completed CONSORT checklist must be included with all submissions.

Clinical trial registration	CIR287(ClinicalTrials.gov NCT02021968) as reported previously (Kirkpatrick etal, Science Translational Medicine, 2016)
Study protocol	Study Protocol CIR287 has been provided
Data collection	A total of 48 subjects were enrolled under study protocol CIR287 (ClinicalTrials.gov NCT02021968) between 11 November 2013 and 25 February 2014. Two groups of 24 subjects.Two groups of twenty-four subjects (12 TV003 recipients and 12 placebo recipients) were tested at sites in Baltimore, MD and Burlington, VT.
Outcomes	The primary efficacy endpoint of the study was the protection afforded by the vaccine against viremia induced by the challenge virus rDENV2Δ30. The incidence, magnitude and duration of challenge virus viremia at study day 180 in subjects who received TV003 on study day 0 was compared to those who received placebo on study Day 0. Secondary endpoints included protection against rash and neutropenia. The proportion of subjects who received TV003 and developed rash/neutropenia following challenge were compared to the proportion of subjects who received placebo and developed rash or neutropenia follwing challenge.
